# Type I collagen fibrils: an inducer of invadosomes

**DOI:** 10.18632/oncotarget.5804

**Published:** 2015-09-23

**Authors:** Julie Di Martino, Violaine Moreau, Frederic Saltel

**Affiliations:** Institut National de la Santé et de la Recherche Médicale and Université de Bordeaux, U1053, Bordeaux, France

**Keywords:** type i collagen, invadosomes, extracellular matrix, podosomes, invadopodia

Cell-extracellular matrix (ECM) interaction controls a multitude of biological and cellular processes such as adhesion, migration, invasion, gene activation, differentiation and proliferation. ECM constitutes a complex and dynamic structure; indeed there are a lot of molecular components able to participate to the ECM formation. We could notably mention collagens, laminins or vitronectin. The ECM composition and functions depend on the tissue, its age and eventually the pathology. Depending on the situation the ECM acts as a support for cells or can represent a physical barrier between different compartments, such as the basement membrane between blood and stroma.

The complexity of interaction between cells and ECM is increased by the variety and the modulation of matrix receptors present at the cell surface. There is a constant communication between cells and the ECM. If ECM was studied for a long time, we have to better understand molecular pathways involved in cell-matrix interaction. In response to modification of its microenvironment, cells can remodel and modify the composition and the physical properties of its surrounding ECM.

Several cell types are able to degrade the ECM to get across matrix barriers and to migrate through connective tissues. At the cellular level, cells form actin-based structures involved in matrix degradation named invadosomes. Invadosomes is a global term enclosing podosomes in normal cells and invadopodia in cancer cells. Invadosomes can be self-organized into different shapes: dots, aggregates or rosettes. Invadosomes molecular complex is composed of several proteins controlling their formation such as actin-binding proteins, actin nucleators (N-WASP or Arp2/3), Rho-GTPase and adaptors. Recently, we established a minimal molecular signature of invadosomes composed by the Rho-GTPase Cdc42 and the adaptor Tks5 [1]. The invadosome degradation activity is driven by metalloproteinases (MMPs) such as MMP2 and MT1-MMP.

Type I collagen was known for a long time to activate the couple MMP2-MT1-MMP. Type I collagen is a very abundant matrix component in physiology. Type I collagen fibrils are present in physiological conditions in a lot of tissues such as skin, tendon and bone. However, its abnormal accumulation is observed in different pathologies such as fibrosis, osteopetrosis and atherosclerosis. Moreover in some cancers, type I collagen fibrils increase is correlated with a poor prognosis and metastasis formation.

All these data suggest that there is a physiological and a pathological organization of type I collagen, or that cells do not respond similarly to type I collagen in pathological conditions. So, we decided to investigate the impact of type I collagen fibrils into invadosome formation and activity. A lot of cells are able to form invadosomes in constitutive condition or after stimulation such as endothelial cells, smooth muscle cells, and hematopoietic cells. Various inducers are already known to promote invadosome formation like cell differentiation, growth factors (EGF, VEGF), pharmaceutical agents (phorbol esters) or activation of different pathways (Src kinase, Rho-GTPases). We determined that type I collagen fibrils are a universal and potent inducer of invadosomes in all tested cells. It triggers their formation in cell devoid of these structures. In cells exhibiting invadosomes, we noticed a linear reorganization of the structures along the type I collagen fibrils, associated with an increase of their degradation activity. Consequently, we named these structures linear invadosomes [2]. Since, other authors confirmed the existence of this invadosome organization and the impact of type I collagen in invadosome activity [3-5]. In a recent study, we established that the discoidin receptor 1 (DDR1) is the receptor involved in linear invadosome formation via a Cdc42-Tuba pathway [6]. DDR1 belongs to a family of tyrosine kinase receptor including DDR1 and DDR2 (DDRs). These ubiquitous receptors are able to interact with collagen fibrils and participate to different cellular processes such as cell adhesion and migration.

DDRs can induce different signaling pathways including MAP kinase and PI-3 kinase/Akt [7]. DDRs play a role in embryo development and also in immune system. Recently, DDRs were involved in various diseases beside cancer, such as atherosclerosis, lung and liver fibrosis, renal injury, wound healing and osteoarthritis. In these different pathologies, type I collagen is also deregulated, for example it is accumulated during fibrosis. In addition, collagen fibrils are modified during these pathologies. So, it will be crucial to fully understand the relationship between DDRs and type I collagen in the establishment of these diseases.
